# Computational investigation of a covalent triazine framework (CTF-0) as an efficient electrochemical sensor†

**DOI:** 10.1039/d1ra08738j

**Published:** 2022-01-31

**Authors:** Sehrish Sarfaraz, Muhammad Yar, Muhammad Ans, Mazhar Amjad Gilani, Ralf Ludwig, Muhammad Ali Hashmi, Masroor Hussain, Shabbir Muhammad, Khurshid Ayub

**Affiliations:** Department of Chemistry, COMSATS University, Abbottabad Campus KPK Pakistan 22060 khurshid@cuiatd.edu.pk +92-992-383591; Department of Chemistry, University of Agriculture Faisalabad 38000 Faisalabad Pakistan; Department of Chemistry, COMSATS University Islamabad, Lahore Campus 54600 Pakistan; Universität Rostock, Institut für Chemie, Abteilung für Physikalische Chemie Dr.-Lorenz-Weg 1 18059 Rostock Germany; Leibniz-Institut für Katalyse an der Universität Rostock Albert-Einstein-Strasse 29a 18059 Rostock Germany; Department of Chemistry, Division of Science & Technology, University of Education 54770 Lahore Pakistan; Department of Data Science, Ghulam Ishaq Khan Institute of Engineering Sciences and Technology Topi KPK Pakistan; Department of Chemistry, College of Science, King Khalid University P. O. Box 9004 Abha 61413 Saudi Arabia

## Abstract

In the current study, a covalent triazine framework (CTF-0) was evaluated as an electrochemical sensor against industrial pollutants *i.e.*, O_3_, NO, SO_2_, SO_3_, and CO_2_. The deep understanding of analytes@CTF-0 complexation was acquired by interaction energy, NCI, QTAIM, SAPT0, EDD, NBO and FMO analyses. The outcome of interaction energy analyses clearly indicates that all the analytes are physiosorbed onto the CTF-0 surface. NCI and QTAIM analysis were employed to understand the nature of the non-covalent interactions. Furthermore, SAPT0 analysis revealed that dispersion has the highest contribution towards total SAPT0 energy. In NBO analysis, the highest charge transfer is obtained in the case of SO_3_@CTF-0 (−0.167 e^−^) whereas the lowest charge transfer is observed in CO_2_@CTF-0. The results of NBO charge transfer are also verified through EDD analysis. FMO analysis revealed that the highest reduction in the HOMO–LUMO energy gap is observed in the case of O_3_ (5.03 eV) adsorption onto the CTF-0 surface, which indicates the sensitivity of CTF-0 for O_3_ analytes. We strongly believe that these results might be productive for experimentalists to tailor a highly sensitive electrochemical sensor using covalent triazine-based frameworks (CTFs).

## Abstract 2

In the current study, a covalent triazine framework (CTF-0) was evaluated as an electrochemical sensor against industrial pollutants *i.e.*, O_3_, NO, SO_2_, SO_3_, and CO_2_.

## Graphical abstract

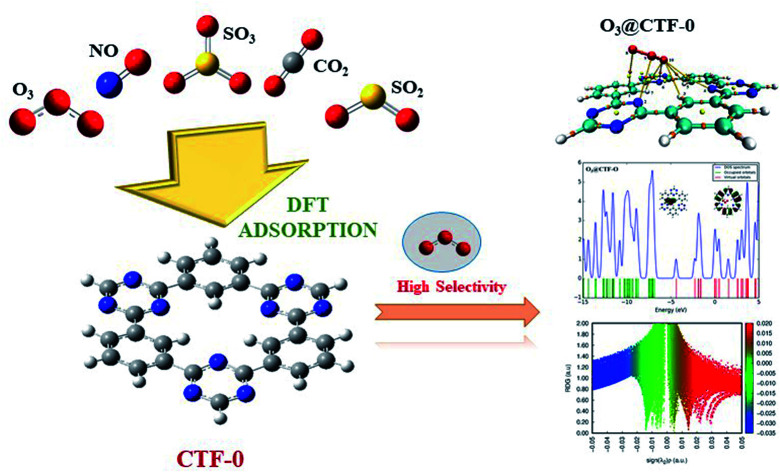

## 1. Introduction

Over the past few years, detection of harmful gases and their appropriate monitoring has gained much attention because these gases are extensively released during numerous industrial and domestic activities.^1^ Therefore, the development of efficient, reliable, and low-cost gas sensors has gained great interest, stretching from industrial to medicinal applications. Seriously harmful gases that are toxic to human health are nitric oxide (NO), carbon dioxide (CO_2_), ozone (O_3_), sulphur dioxide (SO_2_), *etc*.^2^ CO_2_ emission generally occurs as a result of combustion of fossil fuels, and it actively participates in global warming.^3–5^ Moreover, SO_2_ and SO_3_ are the major contributors to air pollution, mainly released during oil and coal burning.^6,7^ On the other hand, emission of ozone (O_3_) targets respiratory tissues and ocular mucosa, thus it has been investigated as the most threatening air pollutant.^8–10^ Hence, the development of highly sensitive, facile, and low-cost gas sensors is of great interest.

Well-organized nano porous materials have gained remarkable attention because of their abundance, exceptional sensing properties and potential applications.^11^ Several nano-porous frameworks have been constructed over the decade such as covalent organic frameworks (COFs), zeolites, and metal–organic frameworks (MOFs). In the past few years, COFs became the center of attention for researchers, due to their highly porous surface, structural properties, physical and chemical durability as well as exhibiting strong covalent bonding.^12–14^ Covalent triazine-based frameworks (CTFs) are considered as a novel promising class of organic porous crystalline materials, first prepared by Thomas *et al.* in 2008. CTFs can be synthesized by trimerization reaction of aromatic nitrile in molten ZnCl_2_ under ionothermal conditions. CTF is a promising class of organic crystalline materials in which their building units are linked through strong covalent bonding to generate 2D and 3D porous structures. Principle modular design of CTFs greatly benefits them to achieve chemical stability, controlled porosity, and valuable adsorption properties.^15–17^

CTFs have various unique properties, for example, the presence of aromatic linkage (C=N) in triazine unit endow CTFs with higher chemical stability, heteroatom effect (HAE) and rich nitrogen contents add value for their applications in practical world.^18^ CTFs have numerous active sites and large π–π stacking, which make them exceptionally potential candidate to acquire some promising characteristics as imparted by graphitic carbon nitride or N-doped graphene surfaces.^19^ These exceptional characteristics enable CTFs for a number of applications including photocatalysis,^20^ solar cells,^25–28^ energy storage,^29^ degradation of organic pollutants,^30^ heterogeneous catalysis,^31^ and electrochemical sensors.^31–35^

In electrochemical sensors, the use of CTFs has attained growing interest in recent years. Novel electrochemical platform for sensing and biosensing based on CTF was reported by Xu *et al.*^36^ Zhang *et al.* reported electrochemical senor based on CTF for the detection of lead ions (Pb^2+^), which displayed a strong response against Pb^2+^ even when the concentration is in nM.^37^ These key applications of CTF suggested that the conjugated triazine rings can play a same role in sensing as played by N-doped graphene and other related materials.^38^

These findings motivated us to explore the wider applications of CTF based electrochemical sensor for the determination of greenhouse and industrial gases. CTF-0 surface was selected due to its large π–π stacking, higher thermal stability and high nitrogen content.^39,40^ The 2D CTF-0 surface provides electron rich porous cavity due to higher nitrogen content which helps in adsorption of analytes.^41^ DFT calculations are performed to evaluate the adsorption studies of industrial gases on the surface of CTF-0. Therefore, we have designed a theoretical study for the detection of five different analytes such as O_3_, NO, SO_2_, SO_3_, and CO_2_ on CTF-0. The adsorption behavior of selected analytes, selectivity and sensitivity of CTF-0 surface is investigated through simple optimization and geometry analysis, HOMO–LUMO gap, and charge transfer through natural bond orbital (NBO) analysis. The nature of interactions between analytes and surface are determined by symmetry adapted perturbation theory (SAPT0), non-covalent interaction (NCI), electron density differences (EDD), and quantum theory of atom in molecule (QTAIM) analysis.

## 2. Computational methodology

Gaussian 09 software^42^ is used for the geometry optimization of complexes (analytes@CTF-0) at M05-2X/6-31G++(d,p) level of theory without symmetry constraints. Optimized geometries were visualized by using GaussView 5.0 package. Various orientations of analytes@CTF-0 were computed to get the most stable geometry. The lowest energy configuration is the most stable one and therefore considered for subsequent calculations. Other properties such as frontier molecular orbital (FMO), band gap, natural bond orbital (NBO) and interaction energies were also evaluated at the same level of theory. The interaction energies for each analyte@CTF-0 complex were calculated by following formula given:

1 Δ*E* = *E*_complex_ − (*E*_analyte_ + *E*_CTF-0_)

*E*_complex_, *E*_analyte_ and *E*_CTF-0_ denote the energies of complex, analyte, and surface, respectively.^43^

Electronic properties were explored *via* Frontier Molecular Orbital (FMO) analysis and density of states (DOS) analysis. The electronic properties are important to understand the characteristics of sensors such as selectivity, sensitivity, conductivity, and resistivity.^44^ Higher conductivity is associated with decreased HOMO–LUMO gaps while enhanced HOMO–LUMO gap reflect higher resistivity.^45^ DOS analysis was performed to get insight into sensor mechanism by evaluating the number of available energy states for electron in a given energy level.^46^ DOS spectra are generated by GaussSum software.^47^ Electronic properties are further elaborated through natural bond analysis (NBO) to evaluate the transfer of charge between analytes and surface (CTF-0).^48^

NCI analysis is mainly employed to distinguish between steric repulsion, electrostatic forces and van der Waals interactions. Non-covalent interactions are important to estimate the adsorption behavior of analyte on surface; therefore, it is essential to evaluate them precisely.^49^ The NCI analysis show the relationship between electron density (*ρ*) and reduced density gradient (RDG) by the following equation:^50^2
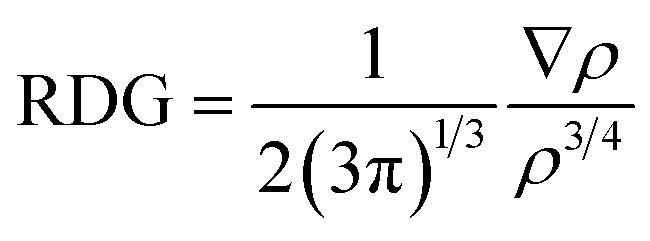


3D plot was used for visual analysis of attractive and repulsive interactions. The scheme of NCI isosurfaces gives insight into the type of interactions present *i.e.*, red color indicates the presence of steric repulsion whereas blue and green parts represent the strong and weak interactions, respectively.^51,52^ The 2D NCI graphs can be obtained by plotting RDG (a. u.) *versus* the product of the sign of second eigenvalue and the density (sign(*λ*_2_)*ρ* (a. u.)). The colored maps in the 2D NCI graph correspond to the same colors of isosurfaces enclosed in 3D plot.^53^ For NCI plots, Multiwfn 3.7 (ref. ^54^) and VMD^55^ software were employed.

SAPT0 analysis was employed to evaluate total interaction energies of complexes (analytes@CTF-0). Four main descriptors are used in the components of SAPT0 analysis and are normally given as: electrostatic (Δ*E*_elstat_), exchange (Δ*E*_exch_), dispersion (Δ*E*_disp_), and induction (Δ*E*_ind_).^56^ The equation for Δ*E*_int_ in SAPT0 analysis is given below:

3 Δ*E*_int_ = Δ*E*_exch_ + Δ*E*_elstat_ + Δ*E*_ind_ + Δ*E*_disp_

PSI4 software was employed to carry out SAPT0 analysis.^57^

Non-covalent interactions were further examined *via* QTAIM analysis. The nature of interactions in QTAIM analysis is evaluated by following parameters: Laplacian of electron density (∇^2^*ρ*), electron charge density (*ρ*), potential energy density *V*(*r*), Lagrangian kinetic energy *H*(*r*), energy density (*H*(*r*)), and *E*_int_. QTAIM analysis is helpful for calculating non-covalent interactions between the fragments from bond critical points.^58^ Electron Density Difference (EDD) analysis is used to explore the electron-transfer behavior of analytes during adsorption and to examine the type of nonbonding interactions between analytes and surface.

## 3. Results and discussions

### 3.1 Geometric optimization

**Fig. 1:**
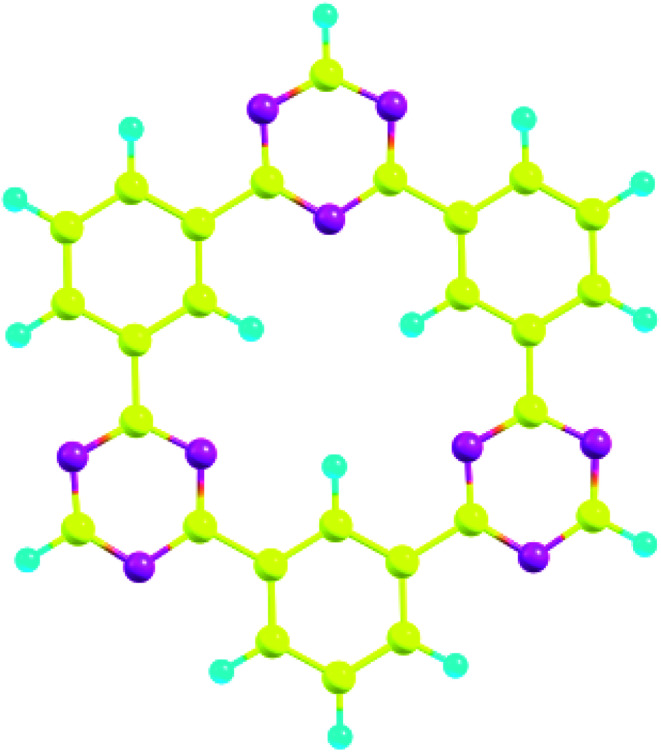
CTF-0 optimized geometry at M05-2X/6-31G++(d,p) level of theory, magenta color for N-atom, yellow denotes C-atom, and turquoise blue for H-atoms.

CTFs are special organic materials with covalent bonding between organic building blocks, which give the 2D and 3D porous structure.^34^ CTFs have gained special attentions due to the availability of numerous active sites, rich nitrogen content, and π linkage among benzene and triazine ring.^30,59^ These characteristics make CTFs a potential candidate for various applications such as photocatalysis,^60^ electrochemical sensors,^61–63^ and degradation of organic pollutants.^64,65^ CTFs are mainly synthesized by trimerization reaction of carbonitriles to produce triazine rings.^66^ The structure of CTF-0 consists of alternative triazine and benzene rings (see Fig. 1). The optimized geometry of CTF-0 reveals that there are three types of bonds based upon bond length and connectivity; C–N bonds with bond length of 1.34 Å while other two bonds are C–C (1.39 Å and 1.47 Å).^67^ Electron rich nitrogen atoms, present in CTF-0 can strongly interact with electron deficient sites of analytes, and hence, resulted in perturbation of electronic structure which provide basis for sensing applications of CTF.^68^

**Fig. 2:**
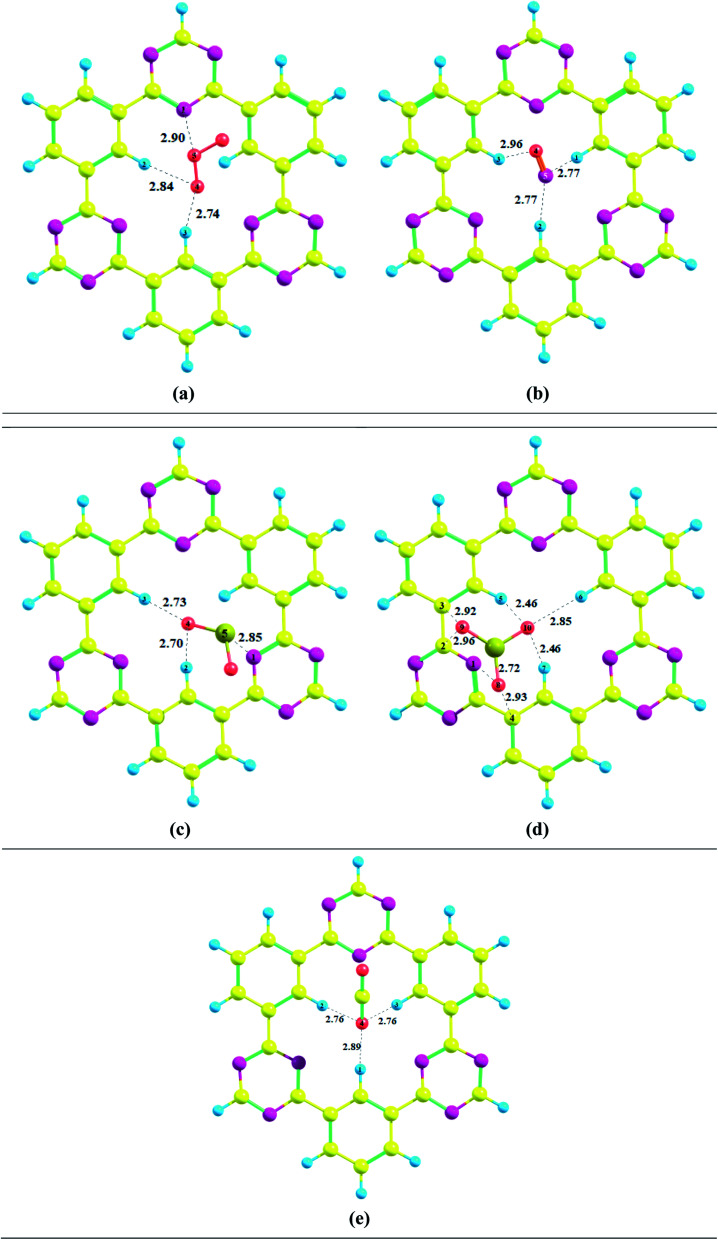
Optimized geometries of selected analytes at CTF-0 surface (top view) at M05-2X/6-31G++(d,p) level of theory. In analytes, pink color indicates the presence of O-atom, mustard yellow presents S-atom, magenta color for N-atom, and yellow denotes C-atom. (a) O_3_@CTF-0, (b) NO@CTF-0, (c) SO_2_@CTF-0, (d) SO_3_@CTF-0 and (e) CO_2_@CTF-0.

Several possible orientations were considered for each analyte on the surface of CTF-0 to get the most stable configuration of analyte@CTF-0 complexes (Fig. S1†). The most stable geometries for each analyte@CTF-0 complex was considered for further analysis and are shown in Fig. 2. In the current study, we have selected five different analytes and checked the sensitivity and selectivity of CTF-0 surface against these analytes. For the sake of convenience, we named the complexes as O_3_@CTF-0, NO@CTF-0, SO_2_@CTF-0, SO_3_@CTF-0, and CO_2_@CTF-0 for ozone, nitric oxide, sulfur dioxide, sulfur trioxide, and carbon dioxide adsorbed on the surface of CTF-0, respectively.

Interaction energy results reveals that the most stable geometry is observed for SO_3_@CTF-0 among the selected complexes because of higher number of possible interactions between SO_3_ and atoms of CTF-0 surface, and smallest interaction distance (see Table 1). The interaction energy (*E*_int_) trend of studied complexes is SO_3_ −0.56 eV (−13.00 kcal mol^−1^) > SO_2_ −0.30 eV (−7.05 kcal mol^−1^) > O_3_ −0.23 eV (−5.44 kcal mol^−1^) > CO_2_ −0.22 eV (−5.03 kcal mol^−1^) > NO −0.16 eV (−3.65 kcal mol^−1^). For physical adsorption, interaction energies must be less than 1 eV and it has been extensively reported in literature. Thus, species with interaction energy <1 eV are considered to be physiosorbed.^69–71^ Small interaction energy (eV) values expresses that all the studied analytes are physiosorbed on the surface (CTF-0).

**Table 1:** Intermolecular distance (Å), interaction energies (kcal mol^−1^) for analytes@CTF-0 complexes at M05-2X/6-31G++(d,p) level of theory

Analytes@CTF-0			
Analyte	Intermolecular bond	Bond length	*E*_int_
O_3_	O5–N1	2.90	−5.44
	O4–H2	2.84	
	O4–H3	2.74	
NO	O4–H3	2.96	−3.65
	N5–H1	2.77	
	N5–H2	2.77	
SO_2_	S5–N1	2.85	−7.05
	O4–H2	2.70	
	O4–H3	2.73	
SO_3_	O10–H5	2.46	−13.00
	O10–H6	2.85	
	O10–H7	2.46	
	O9–H2	2.96	
	O9–H3	2.92	
	O8–N1	2.72	
	O8–H4	2.93	
CO_2_	O4–H1	2.89	−5.03
	O4–H2	2.76	
	O4–H3	2.76	

For O_3_@CTF-0 complex, the least interaction distance of 2.76 Å is observed for O4–H3 bond. The corresponding interaction energy for O_3_@CTF-0 complex is −5.44 kcal mole^−1^. For NO@CTF-0 complex, the least interaction distance of 2.77 Å is obtained for N5–H1 and N5–H2 bonds. The optimized geometry of NO@CTF-0 complex presents the parallel orientation of NO analyte over the CTF-0 surface (Fig. 2). In SO_2_ analyte, one O-atom is oriented towards CTF-0 surface while other O-atom is oriented away from the surface. The most stable conformation of SO_2_@CTF-0 complex shows three strong interactions, (O4–H2) with an interaction distance of 2.70 Å followed by (O4–H3) and (S5–N1) with interaction distances of 2.73 and 2.85 Å, respectively. The highest number of interactions observed in case of SO_3_@CTF-0 complex with the least interaction distance of 2.46 Å for (O10–H5). While highest interaction distance is 2.96 Å for O9–H2 bond. Order of interacting distance is also justified from the interaction energies trend, *e.g.*, the lowest interaction distance 2.46 Å is observed for SO_3_@CTF-0 complex with the interaction energy of −13.00 kcal mol^−1^. In SO_3_@CTF-0 complex, highest interaction energy might be due to the presence of strong hydrogen bonding between O-atoms of SO_3_ analyte and H-atoms of surface. Three electron rich oxygen atoms present in SO_3_ are responsible for the highest interaction energy and lowest interaction distance. On the other hand, the least interaction energy is obtained for NO that is −3.65 kcal mol^−1^ (see Table 1).

The interaction energies with other functionals such as B3LYP-D3 and M06-2X are also calculated and the obtained results are compared with the results of M05-2X (see Table 2). The same trend of interaction energies is observed with different functionals. For example, the highest interaction energies are observed for SO_3_@CTF-0 complex and the lowest interaction energies are seen for NO@CTF-0 complex.

**Table 2:** Comparison of interactions energies (kcal mol^−1^) of studied complexes calculated at three different functionals

*E*_int_ for studied analytes@CTF-O complexes			
Analytes@CTF-0	M05-2X	M06-2X	B3LYP-D3
O_3_@CTF-0	−5.44	−6.29	−6.40
NO@CTF-0	−3.65	−3.89	−3.82
SO_2_@CTF-0	−7.05	−8.48	−9.90
SO_3_@CTF-0	−13.00	−14.99	−16.70
CO_2_@CTF-0	−5.03	−4.96	−5.84

### 3.2 Non-covalent interactions (NCI) analysis

**Fig. 3:**
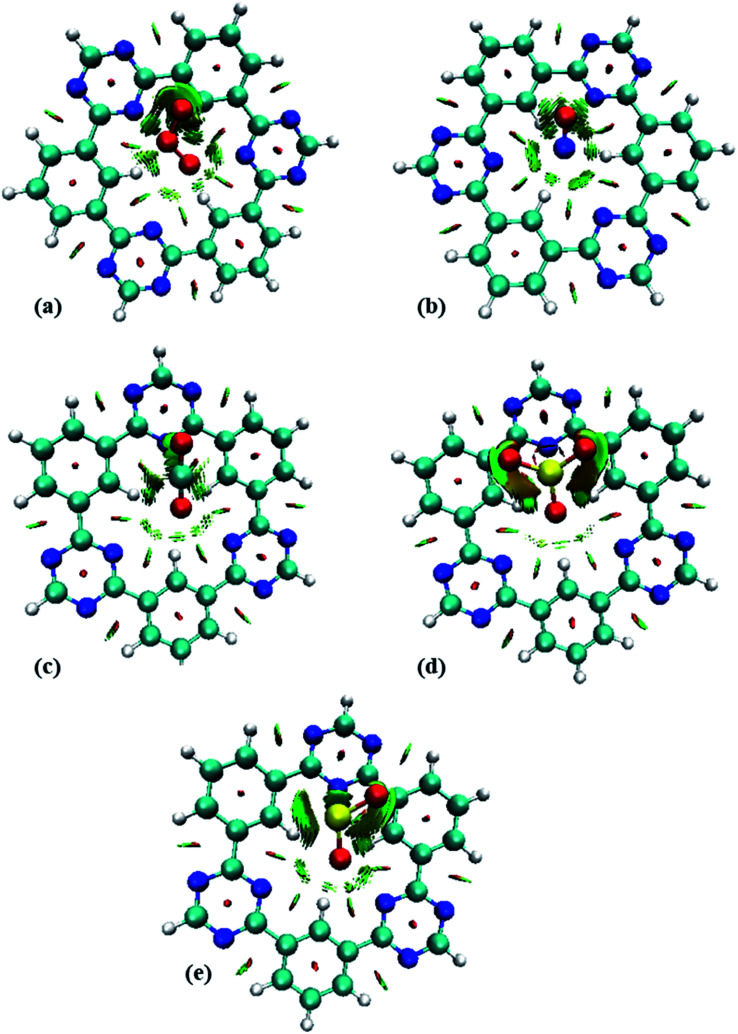
NCI analysis of analytes@CTF-0 with 3D isosurfaces: (a) O_3_@CTF-0, (b) NO@CTF-0, (c) CO_2_@CTF-0, (d) SO_3_@CTF-0 and (e) SO_2_@CTF-0 complex at isovalue of 0.5 a.u. here blue color presents strong electrostatic interactions (hydrogen bonding), green color shows weak interactions (van der Waal's forces), and red color indicates repulsion forces.

To get insight into the type of non-covalent interactions between analytes and CTF-0 in real space, NCI analysis was carried out on the most stable conformations of analyte@CTF-0 complexes. The 3D isosurfaces and 2D-NCI graphs for studied complexes (analytes@CTF-0) are presented to identify the nature of attractive and repulsive forces between analytes and surface. In 3D isosurfaces, the nature of non-covalent interactions was examined on the basis of three colors (Fig. 3). Green color presents weak forces mainly London dispersion interactions and blue color indicates strong interaction such as hydrogen bonding.^72^ Whereas red color shows the steric repulsion present between interacting species.^73^ The isovalue of 0.5 a.u. is used to examine the 3D isosurfaces. The thickness of isosurfaces is directly related with the strength of nonbonding interactions *i.e.*, the thicker patches indicate strong interactions, whereas stippled patches show weak interactions.

**Fig. 4:**
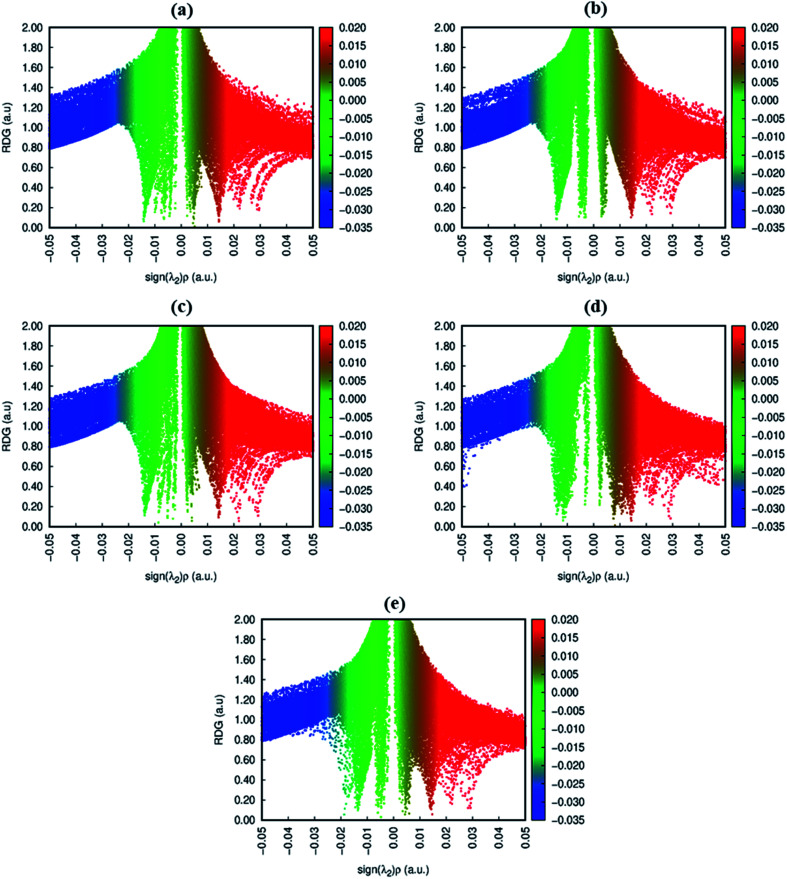
RDG 2D spectra of studied complexes: (a) O_3_@CTF-0, (b) NO@CTF-0, (c) CO_2_@CTF-0, (d) SO_3_@CTF-0 and (e) SO_2_@CTF-0 complex.

The 3D and 2D NCI plots of analytes@CTF-0 complexes are shown in Fig. 3 and 4, respectively. The appearance of green spikes in 2D-NCI plots of studied complexes indicate the presence of non-covalent weak interactions between analytes and CTF-0 on *X*-axis (*λ*_2_)*ρ* 0.00 to −0.015 a.u. are present at the center of benzene and triazine ring of CTF-0, which is the clear indication of steric repulsion presence in the nuclei rings. Furthermore, larger green isosurfaces are observed in case of SO_3_@CTF-0 and SO_2_@CTF-0 complexes in 3D plots, which show that these complexes are more stable as compared to the rest of the complexes.

### 3.3 QTAIM analysis

Bader's quantum theory of atoms in molecules (QTAIM) is mainly used to examine the non-covalent interactions in various molecular systems. QTAIM analysis is the best tool for analyzing intermolecular nonbonding interactions *i.e.*, ionic interactions, van der Waals forces, and hydrogen bonding. The parameters used to investigate the non-covalent interactions *via* QTAIM analysis at a bond critical point (BCP) of studies complexes are electron density (*ρ*(*r*)), kinetic energy density (*G*(*r*)), potential energy density (*V*(*r*)), total energy density (*H*(*r*)), Laplacian of electron density (∇^2^*ρ*(*r*))^74^ and interaction energy (*E*_int_) of an individual bond. The nature of the nonbonding interactions is characterized through Laplacian of charge density (∇^2^*ρ*(*r*)), whereas the strength of bond at bond critical point (BCP) is evaluated *via* electron density (*ρ*). The nature of bonding can be estimated through individual bond interaction energies, calculated by the Espinosa approach.4
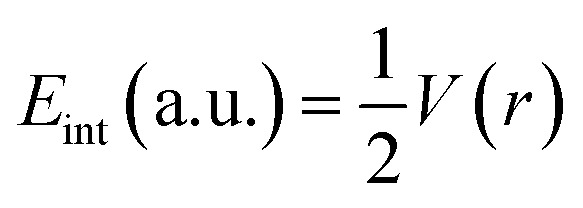


Interaction energy (*E*_int_) values in the range of 3–10 kcal mol^−1^ indicate the presence of hydrogen bonding.^75^

If charge concentrations in Laplacian of electron density (∇^2^*ρ*(*r*)) is less than zero *i.e.*, (∇^2^*ρ*(*r*) < 0) it indicates chemical bonding. On the other hand, if charge concentration is greater than zero *i.e.*, (∇^2^*ρ*(*r*) > 0), it shows weak intermolecular interactions. Change in potential and kinetic energy values resulted in the net rise of molecular energy, and these changes that occur in the bonding regions are calculated by Bader equation given below:5
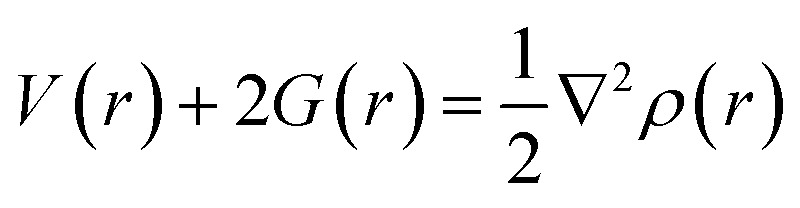


The above equation combines P.E. density *V*(*r*) and K.E. density *G*(*r*) with Laplacian of electron density (∇^2^*ρ*(*r*)). Moreover, energy density *H*(*r*) can be calculated by the formula given below:

6 *G*(*r*) + *V*(*r*) = *H*(*r*)

**Fig. 5:**
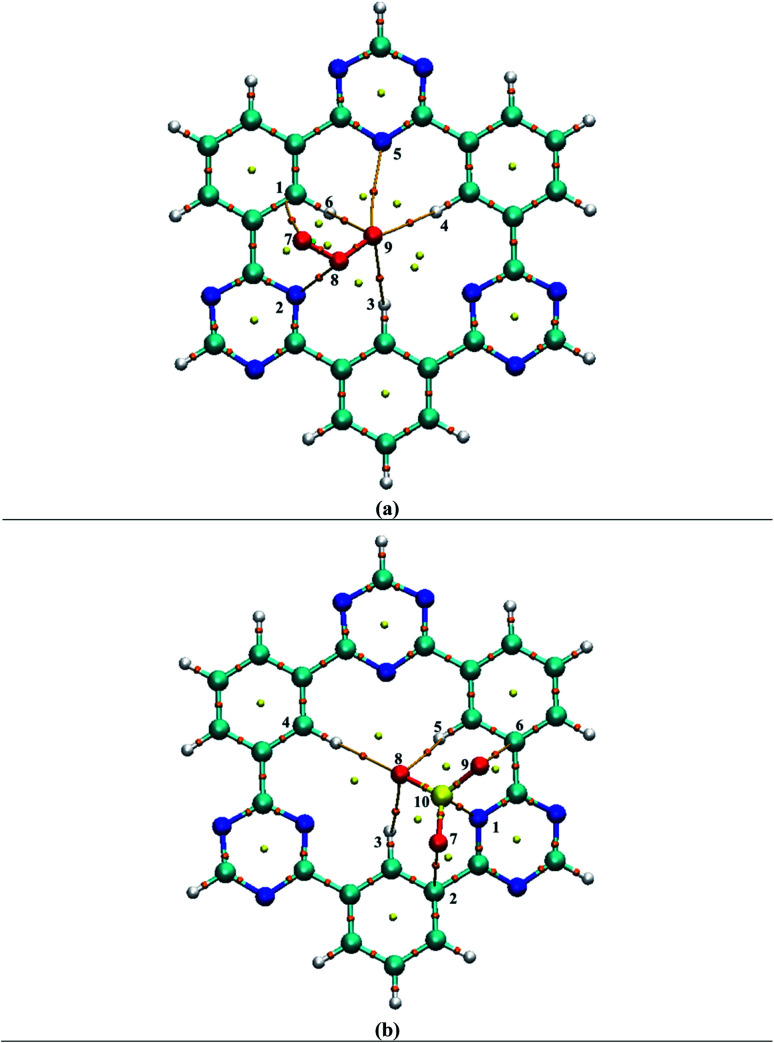
QTAIM analysis results of analytes@CTF-0 complexes *i.e.*, (a) O_3_@CTF-0 complex and (b) SO_3_@CTF-0 complex: bond paths are indicated by lines between analyte and CTF-0 surface, whereas bond critical point (BCPs) are indicated by colored dots.

For weak interactions, the energy density *H*(*r*) must either be zero or less than zero *i.e.*, *H*(*r*) < 0. While the energy density *H*(*r*) greater than zero indicates the appearance of covalent bonding in the region.^76,77^ Furthermore, interatomic interactions can be estimated through the −*V*(*r*)/*G*(*r*) ratio. If the ratio is less than one *i.e.*, −*V*(*r*)/*G*(*r*) < 1, it shows the presence of weak interactions, while the ratio −*V*(*r*)/*G*(*r*) > 2 indicates covalent bonding. Results of topological parameters obtained *via* QTAIM analysis of analytes@CTF-0 are shown in Table 3. The BCPs between analytes and surface (CTF-0) are presented through colored map and are given in Fig. 5.

**Table 3:** Nonbonding interactions study of analytes@CTF-0 complexes *via* QTAIM analysis

Analytes@CTF-0	CTF-0—analyte	*ρ* (a.u.)	∇^2^*ρ* (a.u.)	*G*(*r*) (a.u.)	*V*(*r*) (a.u.)	*H*(*r*) (a.u.)	−*V*/*G*	*E*_int_ (kcal mol^−1^)
O_3_@CTF-0	**H5–O10**	0.004	0.017	0.003	−0.0026	0.0008	0.87	−0.82
	**H3–O10**	0.004	0.017	0.003	−0.0026	0.0008	0.87	−0.82
	**N6–O10**	0.011	0.034	0.008	−0.0071	0.0070	0.88	−2.19
	**H7–O10**	0.007	0.028	0.006	−0.0049	0.0011	0.82	−1.53
	**C1–O8**	0.006	0.021	0.004	−0.0039	0.0006	0.97	−1.22
	**N2–O9**	0.010	0.033	0.007	−0.0070	0.0060	1.00	−2.19
NO@CTF-0	**N5–N7**	0.003	0.011	0.002	−0.0020	0.0005	1.00	−0.63
	**H6–N7**	0.005	0.018	0.004	−0.0027	0.0009	0.67	−0.85
	**N1–N7**	0.003	0.011	0.002	−0.0018	0.0005	0.90	−0.56
	**H2–N7**	0.005	0.018	0.004	−0.0027	0.0009	0.67	−0.85
	**H4–N7**	0.005	0.020	0.004	−0.0031	0.0009	0.77	−0.97
	**N3–O8**	0.004	0.017	0.004	−0.0031	0.0005	0.77	−0.97
SO_2_@CTF-0	**H6–O8**	0.006	0.025	0.005	−0.0039	0.0011	0.78	−1.22
	**N5–O8**	0.003	0.014	0.003	−0.0024	0.0005	0.80	−0.75
	**H4–O8**	0.004	0.018	0.004	−0.0029	0.0009	0.72	−0.91
	**N3–O8**	0.002	0.010	0.002	−0.0014	0.0006	0.70	−0.44
	**C7–O10**	0.004	0.016	0.003	−0.0027	0.0007	0.90	−0.85
	**C2–S9**	0.005	0.021	0.004	−0.0030	0.0012	0.75	−0.94
	**N1–S9**	0.019	0.054	0.012	−0.0112	0.0011	0.93	−3.51
SO_3_@CTF-0	**H4–O8**	0.003	0.014	0.003	−0.0020	0.0008	0.67	−0.63
	**H5–O8**	0.010	0.039	0.009	−0.0074	0.0011	0.82	−2.32
	**H3–O8**	0.010	0.039	0.009	−0.0074	0.0011	0.82	−2.32
	**N1–S10**	0.018	0.052	0.004	−0.0102	0.0011	0.72	−3.20
	**C2–O7**	0.011	0.039	0.009	−0.0080	0.0009	0.89	−2.51
	**C6–O9**	0.011	0.039	0.009	−0.0080	0.0009	0.89	−2.51
CO_2_@CTF-0	**H2–O5**	0.005	0.020	0.004	−0.0031	0.0010	0.77	−0.97
	**H3–O5**	0.003	0.014	0.003	−0.0018	0.0008	0.60	−0.56
	**H4–O5**	0.005	0.020	0.004	−0.0031	0.0010	0.77	−0.97
	**N1–O6**	0.008	0.032	0.007	−0.0056	0.0012	0.80	−1.76

Different BCPs for all complexes are observed which indicate the presence of different types of interactions between analytes and surface. The stable geometry of O_3_@CTF-0 complex reveals six BCPs, which consist of three H–O, two N–O and one C–O bond interactions (see Table 3). The electronic density *ρ*(*r*) values for O_3_@CTF-0 complex are found in the range of (0.004 to 0.011 a.u.) and Laplacian of electron density ∇^2^*ρ*(*r*) values are 0.017 to 0.034 a.u. The highest values of *ρ* (0.011 a.u.) and ∇^2^*ρ* (0.034 a.u.) indicate the presence of strong non-covalent interaction between N6 of CTF-0 and O10 atom of O_3_ analyte. In case of NO@CTF-0 complex, six BCPs are observed out of which three are present in H–N bond (see Fig. S2†). The *ρ*(*r*) values are observed in the range of 0.003 to 0.005 a.u. While ∇^2^*ρ*(*r*) values for the observed BCPs are in the range of 0.011 to 0.020 a.u. and *H*(*r*) values are 0.0005 to 0.0009 a.u. These values indicate medium non-covalent interaction between NO and CTF-0. Seven BCPs are observed in stable geometry of SO_2_@CTF-0 complex. The results indicate that SO_2_ get stabilized on CTF-0 surface *via* two H–O, two N–O, one C–O, one C–S and one N–S types of interactions. The remaining topological parameters such as *G*(*r*), *V*(*r*), *H*(*r*), and −*V*(*r*)/*G*(*r*) values also show the existence of non-covalent interactions.

In case of SO_3_@CTF-0 complex, six BCPs obtained and SO_3_ is stabilized on CTF-0 surface with three H–O, two C–O, and one N–S bonds (Fig. 5). The strongest interaction is observed for N1–S10 bond in SO_3_@CTF-0 complex (see Table 3). For CO_2_@CTF-0 complex, four BCPs are found with three H–O and one N–O bonds. The strongest interaction is observed for N–O bond. Topological values for ∇^2^*ρ*, *ρ*, *H*(*r*), and −*V*(*r*)/*G*(*r*) are 0.032 (a.u.), 0.008 (a.u.), 0.0012 (a.u.), and 0.80 (a.u.), respectively. Individual bond interaction energy (*E*_int_) confirms the absence of hydrogen bonding (strong interactions) in all complexes. The *E*_int_ values lie in the range of −0.44 kcal mol^−1^ to −3.20 kcal mol^−1^ in all analyte@CTF-0 complexes. In QTAIM analysis, all the topological parameters reveal that analyte@CTF-0 complexes are stabilized through non-covalent interactions.

### 3.4 SAPT0 analysis

Symmetry adapted perturbation theory (SAPT0) analysis is carried out to fully quantify and understand the nature of interactions between analytes and CTF-0 surface. SAPT0 analysis consists of four interaction energy contributors *i.e.*, induction (Δ*E*_ind_), exchange (Δ*E*_exch_), electrostatic (Δ*E*_elst_), and dispersion (Δ*E*_disp_). SAPT0 analysis is a very useful tool for understanding the physical nature of non-covalent bonding. The exchange energy (*E*_exch_) part is responsible for repulsive forces between two interacting components *i.e.*, analyte and surface, while *E*_est_, *E*_ind_, and *E*_dis_ play role in attractive forces. The *E*_dis_ component attributed towards weak London dispersion forces. The *E*_elst_ is crucial in stabilizing complexes, while *E*_exch_ component is responsible for destabilizing the complexes.^78^ Sum of all four components *E*_ind_, *E*_exch_, *E*_elst_, and *E*_dis_ give *E*_SAPT0_ (total SAPT0 interaction energy) as presented in eqn (3).^79^

Interaction energy values for SAPT0 analysis of studied analytes@CTF-0 are reported in Table 4. Exchange part (Δ*E*_exch_) of SAPT0 analysis is +ive which shows the existence of repulsive force between the filled orbitals of two interacting components. The results also indicate that majority components of energy in SAPT0 analysis are −ive which presents attractive forces between interacting components *i.e.*, analytes and CTF-0 (see Table 4). Trend of *E*_SAPT0_ for analytes@CTF-0 complexes show an acceptable agreement with the interaction energy results. *E*_SAPT0_ energy values reveals that the highest stabilization energy is obtained for SO_3_@CTF-0 complex whereas the least value is obtained for O_3_@CTF-0, consistent with *E*_int_ results (see Table 1).

**Table 4:** SAPT0 analysis for non-covalent interactions of analytes@CTF-0 complexes (kcal mol^−1^)

Analytes@CTF-0	Δ*E*_elst_	%	Δ*E*_exch_	Δ*E*_ind_	%	Δ*E*_dis_	%	Δ*E*_SAPT0_
O_3_@CTF-0	−3.56	24.71	7.28	−1.13	7.84	−9.72	67.45	−6.57
NO@CTF-0	−1.57	20.02	3.93	−0.31	3.95	−5.96	76.02	−2.71
SO_2_@CTF-0	−9.05	34.83	15.61	−4.19	16.12	−12.74	49.04	−10.38
SO_3_@CTF-0	−51.76	60.48	85.62	−4.39	5.13	−29.43	34.39	−21.51
CO_2_@CTF-0	−3.53	21.67	5.96	−0.71	4.35	−12.05	73.97	−9.33

**Fig. 6:**
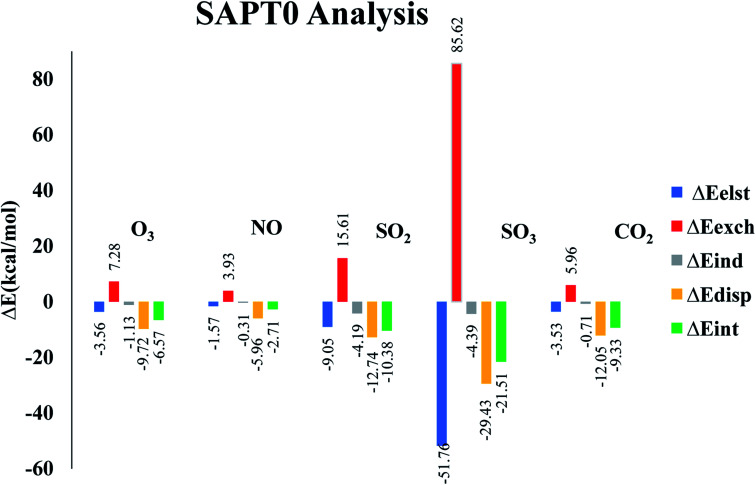
SAPT0 values of selected analytes@CTF-0 graphical representation.

The highest contribution of *E*_exch_ is observed for SO_3_@CTF-0 complex (85.62 kcal mol^−1^) followed by NO@CTF-0, SO_2_@CTF-0, O_3_@CTF-0 and CO_2_@CTF-0 complexes with 3.93, 15.61, 7.28 and 5.96 kcal mol^−1^, respectively (see Fig. 6). The other SAPT0 energy components for O_3_@CTF-0 are −3.56 kcal mol^−1^ (*E*_elst_), −1.13 kcal mol^−1^ (*E*_ind_) and −9.72 kcal mol^−1^ (*E*_disp_). Results clearly show that major stabilizing factor is the dispersion component (67.45%), while electrostatic and induction components contribute 24.71% 7.84%, respectively. Similar energy trend of SAPT0 components is observed for NO@CTF-0 complex. For SO_2_@CTF-0 complex, the contribution of *E*_elst_, *E*_ind_ and *E*_disp_ are −9.05 kcal mol^−1^, −4.19 kcal mol^−1^ and −12.74 kcal mol^−1^, respectively. *E*_disp_ is a dominant factor for SO_2_@CTF-0 complex with 49.04% contribution. The trend of SAPT0 energy components of SO_2_@CTF-0 complex is quite comparable with O_3_@CTF-0 *i.e.*, *E*_disp_ > *E*_elest_ > *E*_ind_. In case of SO_3_@CTF-0 complex, *E*_elest_ is a dominant component with 60.48% (−51.76 kcal mol^−1^) contribution, whereas *E*_disp_ and *E*_ind_ are less dominant with 34.39% and 5.13% contribution towards the total SAPT0, respectively. CO_2_@CTF-0 complex again follows the trend: *E*_disp_ > *E*_elest_ > *E*_ind_ with highest contribution of *E*_disp_ (73.97%), while *E*_elest_ and *E*_ind_ are less significant with 21.67% and 4.35% contribution, respectively.

Overall order of contribution of SAPT0 components towards total *E*_SAPT0_ is *E*_disp_ > *E*_elest_ > *E*_ind_. These SAPT0 component values indicate that the major stabilizing factor is *E*_disp_. The energy trend obtained *via* SAPT0 analysis show an appreciable agreement with NCI and *E*_int_ analysis.

## 4. Electronic properties

### 4.1 Natural bond orbital (NBO) and electron density differences (EDD) analysis

**Fig. 7:**
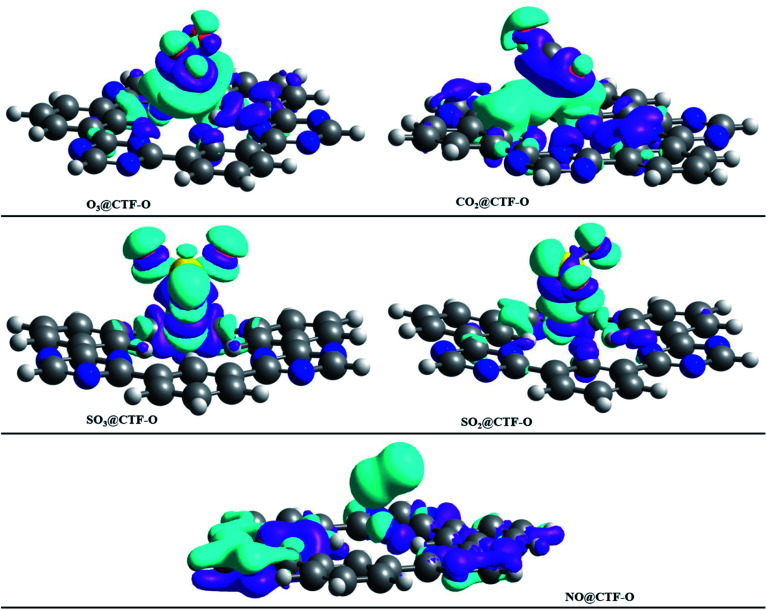
EDD plot of various analytes@CTF-0 complexes: purple color indicates the electron deficiency or electron density depletion, while cyan blue isosurfaces represent the increase in electron density, (isovalue = 0.004 a.u.).

EDD analysis was employed to study the isosurfaces of studied analytes@CTF-0 complexes (see Fig. 7) and their NBO charges are presented in Table 5. The charge accumulation in isosurface of analytes@CTF-0 complexes is presented by cyan blue regions, whereas the purple regions show charge depletion or electron deficiency during adsorption process of analytes on the surface (CTF-0). On complexation, the NBO charge values observed on analytes are: 0.007 e^−^ (O_3_), 0.009 e^−^ (NO), −0.026 e^−^ (SO_2_), −0.167 e^−^ (SO_3_), and 0.002 e^−^ (CO_2_). The NBO values show that in case of SO_2_ and SO_3_, charge is transferred from surface (CTF-0) to analytes, whereas in case of O_3_, NO and CO_2_, the charge is being transferred from analyte to surface. The highest charge transfer in NBO analysis is observed for SO_3_@CTF-0 complex, which might be due to the appearance of strong H-bond between O-atom of SO_3_ and H-atom of CTF-0. Whereas the least charge transfer is observed in case of CO_2_@CTF-0 complex.

**Table 5:** Results of NBO analysis and FMO analysis

Complexes	LUMO (a.u.)	eV	HOMO (a.u.)	eV	HOMO LUMO band gap	NBO (e^−^)
O_3_@CTF-0	−0.126	−3.42	−0.310	−8.45	5.03	0.007
NO@CTF-0	−0.062	−1.70	−0.291	−7.91	6.21	0.009
SO_2_@CTF-0	−0.078	−2.12	−0.310	−8.45	6.33	−0.026
SO_3_@CTF-0	−0.081	−2.20	−0.313	−8.51	6.32	−0.167
CO_2_@CTF-0	−0.063	−1.71	−0.311	−8.46	6.75	0.002
CTF-0	−0.052	−1.42	−0.303	−8.26	6.84	

NBO charges also justify EDD analysis for studied analytes@CTF-0 complexes. In all analytes@CTF-0 complexes, the appearance of green isosurfaces reveal that appreciable charge exchange occurs between analytes and CTF-0 surface.

### 4.2 Frontier molecular orbital (FMO) and density of states (DOS) analysis

The sensing ability of an electrochemical sensor is solely relying on the behavior of its electronic properties on complexation. Frontier molecular orbitals (FMO) analysis is carried out to disclose the sensing ability of CTF-0 surface towards toxic pollutants. Thus, electronic properties are crucial in understanding sensor's selectivity, sensitivity, and conductivity of surfaces (in our case CTF-0) towards toxic pollutants.^44^ Higher conductivity is resulted from decreased HOMO–LUMO gaps whereas higher resistivity resulted from increased energy gap. The HOMO–LUMO energies and band gap (*E*_H–L_) energies (eV) for bare CTF-0 and complexes (analytes@CTF-0) are given in Table 5.

**Fig. 8:**
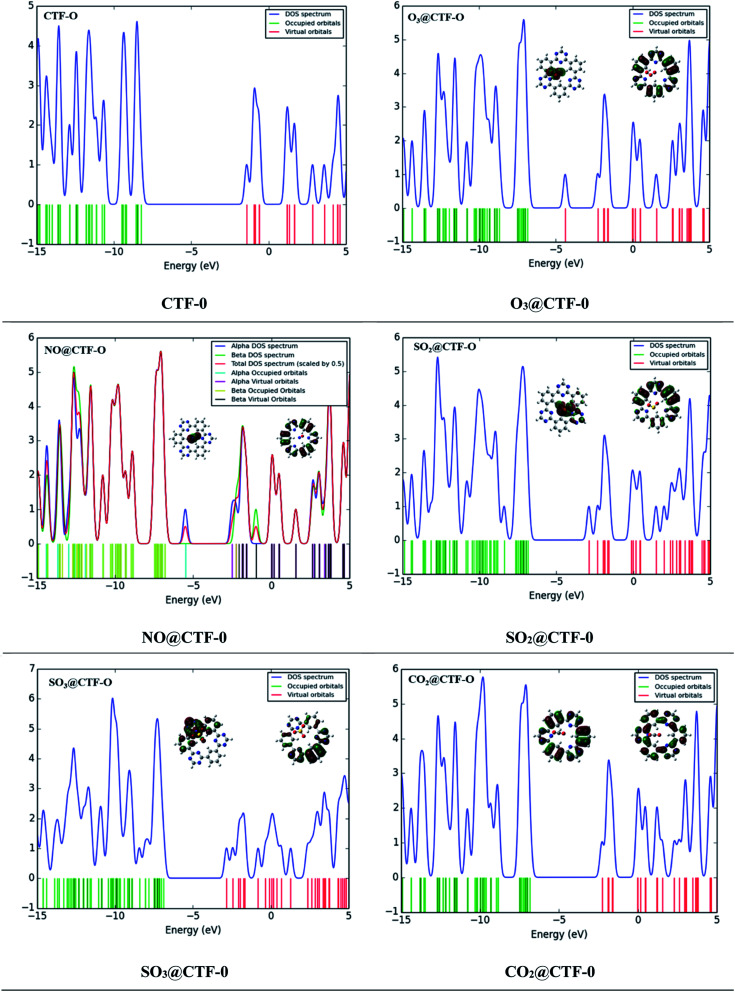
TDOS studies and FMO distribution patterns of the most stable analytes@CTF-0 complexes.

For bare CTF-0 surface, HOMO–LUMO energy gap (*E*_H–L_) is 6.84 eV whereas HOMO–LUMO energy values observed are −8.26 eV and −1.42 eV, respectively. On adsorption of analytes, the reduction in HOMO–LUMO energy gap of studied analytes@CTF-0 are 5.03 eV (O_3_@CTF-0), 6.21 eV (NO@CTF-0), 6.33 eV (SO_2_@CTF-0), 6.32 eV (SO_3_@CTF-0), and 6.75 eV (CO_2_@CTF-0) complexes. Reduction in HOMO–LUMO energy gap (*E*_H–L_) of complex results in enhanced sensing ability of surface. Significant decrease in *E*_H–L_ gap (5.03 eV) has been observed for O_3_@CTF-0 complex. This decrease in *E*_H–L_ gap results in better adsorption of O_3_ analyte on surface, thus making it highly sensitive towards CTF-0. In FMO analysis, HOMO density is mostly localized on analytes, whereas LUMO is observed on CTF-0 surface (see Fig. 8). Electronic excitations result in electron density transfer from analytes to CTF-0 surface, therefore, causes considerable decrease in *E*_H–L_ gaps in case of O_3_@CTF-0 (5.03 eV) complex followed by SO_3_@CTF-0 and SO_2_@CTF-0 complexes.

Change in conduction properties is the most valuable tool to examine the adsorption behavior of toxic pollutants in gas sensors. A strong correlation exists between the macroscopic property with microscopic property (*e.g.*, conductivity) with density of states (DOS).^80–82^ DOS analysis of studied analytes@CTF-0 complexes and bare CTF-0 is carried out comparatively to confirm interaction of analytes with CTF-0 surface upon complexation (see Fig. 8). DOS spectrum of O_3_@CTF-0 reveals the shifting of LUMO from −1.4 eV to −3.42 eV orbital upon complexation. Similarly, in case of other studied complexes, LUMOs are shifted to −1.70, −2.12, −2.20 and −1.71 eV in NO@CTF-0, SO_2_@CTF-0, SO_3_@CTF-0 and CO_2_@CTF-0 complexes, respectively. While *E*_HOMO_ is originally observed at −8.26 eV, while on complexation these are shifted to −8.45, −7.91, −8.45, −8.51 and −8.46 eV in O_3_@CTF-0, NO@CTF-0, SO_2_@CTF-0, SO_3_@CTF-0 and CO_2_@CTF-0 complexes, respectively. This shifting of HOMO–LUMO upon complexation reduces the HOMO–LUMO energy gaps (*E*_H–L_), consequently accredit to higher conductivity and sensitivity.

## 5. Recovery response of sensor

Recovery time of a sensor is an important parameter in determination of its superiority over other electrochemical sensors. Higher recovery time will result in surface poisoning, whereas sensor with very small recovery time does not get appreciable time for interaction. Therefore, an ideal sensor should have suitable recovery response time for adsorption of analyte. Recovery times of CTF-0 were measured at three different temperatures *i.e.*, 298 K, 350 K and 400 K. Theoretically, we have measured recovery time of CTF-0 sensor *via* thermal effect by employing equation from “transition state theory”,^83^^,^^80^ which can be written as:7
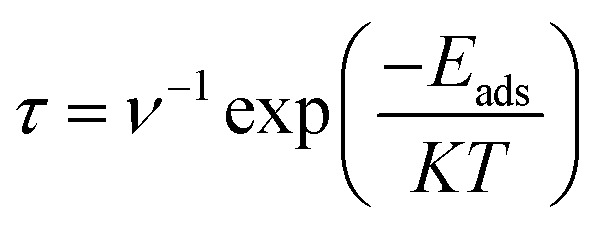


In eqn (7), *τ* denotes recovery time, *E*_ads_ is for interaction energy, *T* is for temperature, *K* denotes Boltzmann constant and *ν* is showing attempt frequency. The value of attempt frequency (*ν*) has already been reported and is 10^12^ s^−1^.^81,82^*K* is constant and its value is 1.99 × 10^−3^ kcal mol^−1^. Greater *E*_ads_ value in negative will prolong the recovery time exponentially (from eqn (7)). The recovery time for studied analytes are calculated at three different temperatures on CTF-0 surface. At room temperature, 9.72 × 10^−9^ s of recovery time is observed for the O_3_ analyte desorption from CTF-0 surface. It is observed that by rising temperature the recovery time further decreases, for example at 350 K and 400 K, the recovery times observed are 2.48 × 10^−9^ s and 9.35 × 10^−10^ s, respectively. Whereas for the desorption of NO, SO_2_, SO_3_, and CO_2_ analytes from CTF-0 at room temperature, the values for recovery time are 4.74 × 10^−10^ s, 1.47 × 10^−7^ s, 3.39 × 10^−3^ s, and 4.87 × 10^−9^ s, respectively. Already reported optimal accumulation times for C_2_N surface were 7.8 × 10^−4^ s and 6.1 × 10^−5^ s at 350 K and 400 K, respectively.^74^ Similarly, in literature, optimal recovery time of 200 s is observed for CNTs-GO surface indicating that the surface had the ability to accumulate the analytes effectively.^83^ The small recovery time values reveal the potential of CTF-0 surface as a fascinating candidate for sensing applications. Additionally, it has been revealed that for all studied analytes rise in temperature result in decline of recovery time (see Table S1†) because the process of desorption gets facilitated by increase in temperature.

## 6. Conclusions

Herein, we explored 2-D surface CTF-0 as an electrochemical sensor against industrial energy analysis clearly indicates that all the analytes are physiosorbed onto the CTF-0 surface. To get further insight into non-covalent interactions, we performed NCI, SAPT0, and QTAIM analysis. In NCI analysis, the appearance of isosurfaces confirms the existence of intermolecular attractive forces between analytes and CTF-0 surface. Furthermore, SAPT0 analysis unveiled that dispersion interactions are dominant, and overall order of contribution of SAPT0 components towards total *E*_SAPT0_ is *E*_disp_ > *E*_elest_ > *E*_ind_. QTAIM analysis further confirms the physisorption of analytes on CTF-0 surface through number of BCPs. The dispersion interactions revealed through the values of *ρ*, ∇^2^*ρ* and *H*(*r*) are in accordance with SAPT0 results. Significant modifications in the electronic properties are observed through FMO analysis for all complexes. The highest decrease in HOMO–LUMO energy gap is observed for O_3_@CTF-0 as compared to bare CTF-0 surface, which indicates the sensitivity of CTF-0 for O_3_ analyte. Moreover, the ability of CTF-0 to detect industrial pollutants is further explored using NBO charge transfer, EDD and DOS analysis. These properties represented that conductivity is improved with band gap reduction upon complexation. These findings propose the CTF-0 as an efficient electrochemical sensor for the detection of industrial pollutants.

## Conflicts of interest

There are no conflicts to declare.

## Supplementary Material

RA-012-D1RA08738J-s001
