# Supplementary material for: Computational investigation of a covalent triazine framework (CTF-0) as an efficient electrochemical sensor
Source: RSC Adv. 2022 Jan 31;12(7):3909–23. doi: 10.1039/d1ra08738j (PMC8981076; doi:10.1039/d1ra08738j)
Supplement: RA-012-D1RA08738J-s001 [file RA-012-D1RA08738J-s001.pdf]

Electronic Supplementary Material (ESI) for RSC Advances.
This journal is © The Royal Society of Chemistry 2022

Computational investigation of covalent triazine framework (CTF-0) as an efficient electrochemical sensor

Sehrish Sarfaraz[a#], Muhammad Yar[a#], Muhammad Ans[b], Mazhar Amjad Gilani[c], Ralf Ludwig[d], Shabbir, Muhammad Ali Hashmi[e] Masroor Hussain[g], & Khurshid Ayub[a]*

[a] Department of Chemistry, COMSATS University, Abbottabad Campus, KPK, Pakistan

22060

[b] Department of Chemistry, University of Agriculture Faisalabad, 38000, Faisalabad,

Pakistan

[c] Department of Chemistry, COMSATS University Islamabad, Lahore campus, 54600, Pakistan

[d] Department of Chemistry, University of Rostock, Dr.-Lorenz-Weg 1, 18059, Rostock, Germany

[e] Department of Physics, College of Science, King Khalid University, Abha, 61413, P.O. Box 9004, Saudi Arabia

[f] Department of Chemistry, University of Education, Attock Campus, Attock, Punjab, 43600, Pakistan

[g] Department of Data Science, Ghulam Ishaq Khan Institute of Engineering Sciences

*Corresponding author.

Tel: +92-992-383591.

E-mail: khurshid@cuiatd.edu.pk (K.A.)

# Sehrish Sarfaraz and Muhmmad Yar have equal contribution for first authorship

<table>
<tr><td colspan="2">O_3@CTF-0</td></tr>
<tr><td>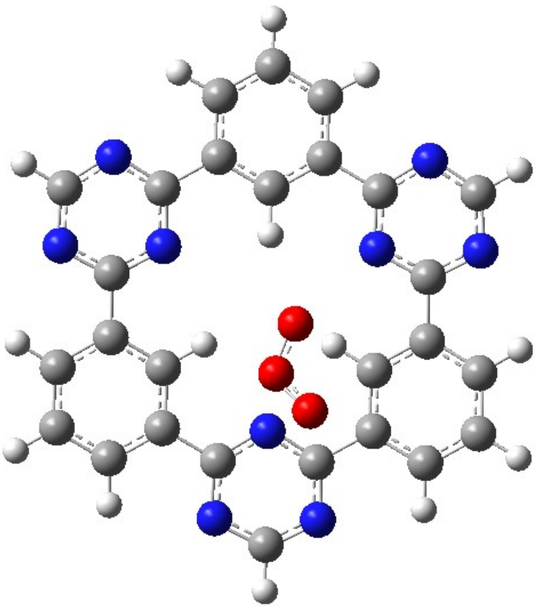
-5.01 kcal/mol</td><td>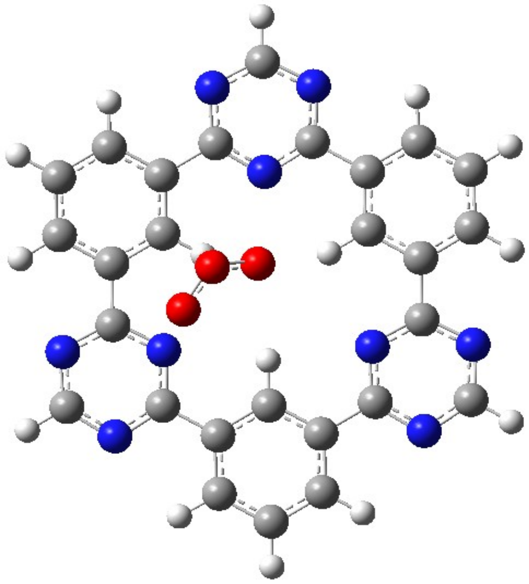
-4.86 kcal/mol</td></tr>
<tr><td colspan="2">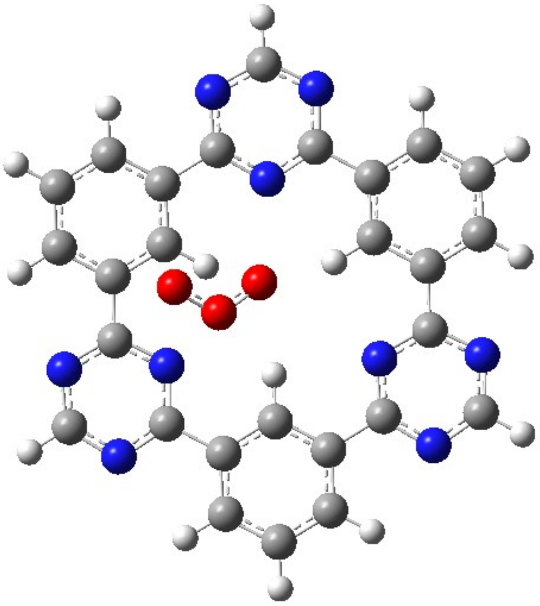
-4.56 kcal/mol</td></tr>
<tr><td colspan="2">NO@CTF-0</td></tr>
</table>

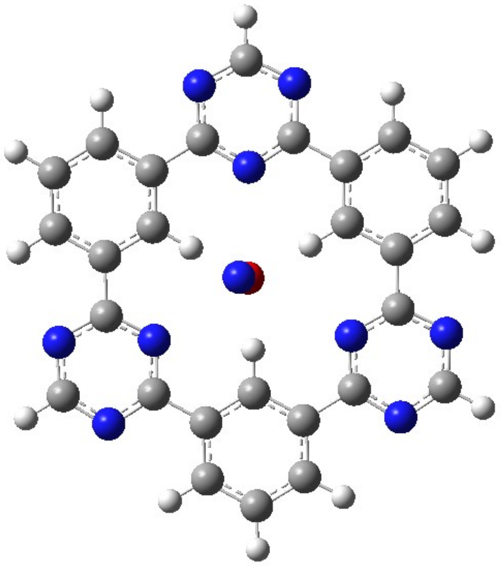 **-3.38 kcal/mol**	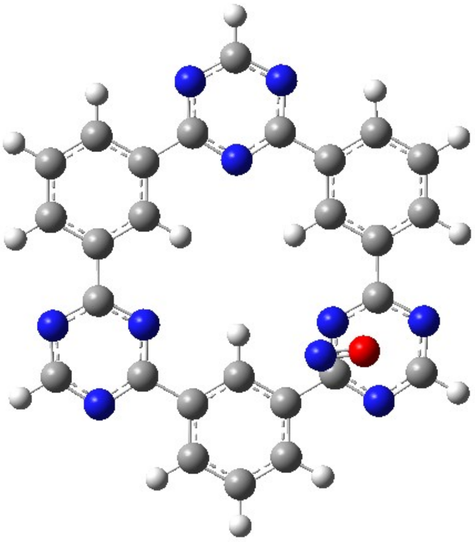 **-3.02 kcal/mol**
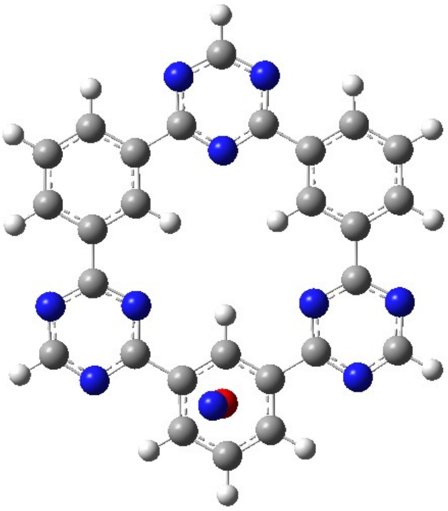 **-2.98 kcal/mol**	
**SO_2@CTF-0**	

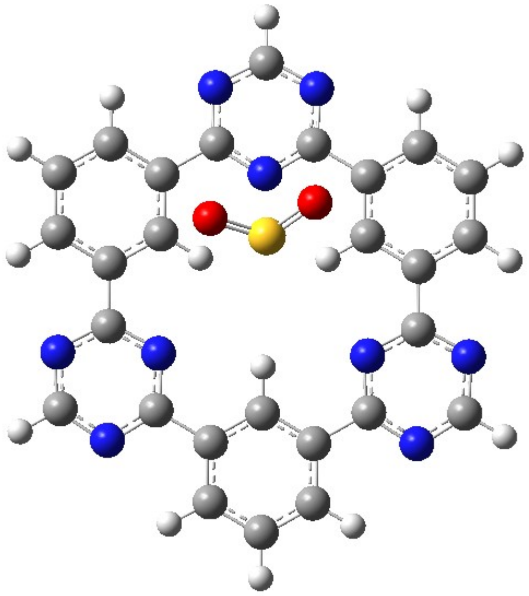<br>**-6.67 kcal/mol**	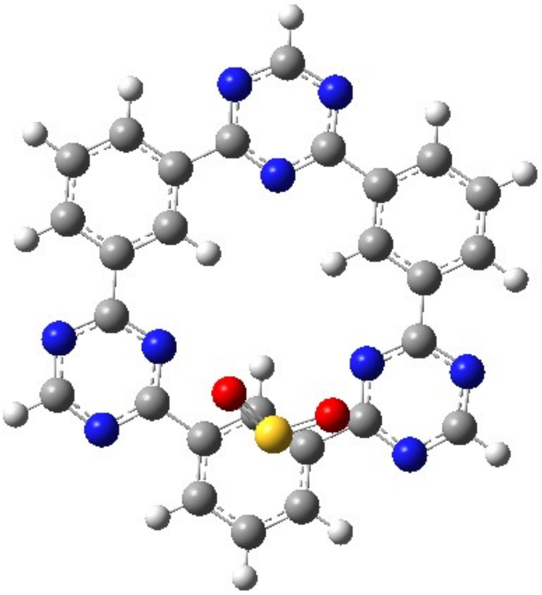<br>**-6.21 kcal/mol**
**SO_3@CTF-0**	
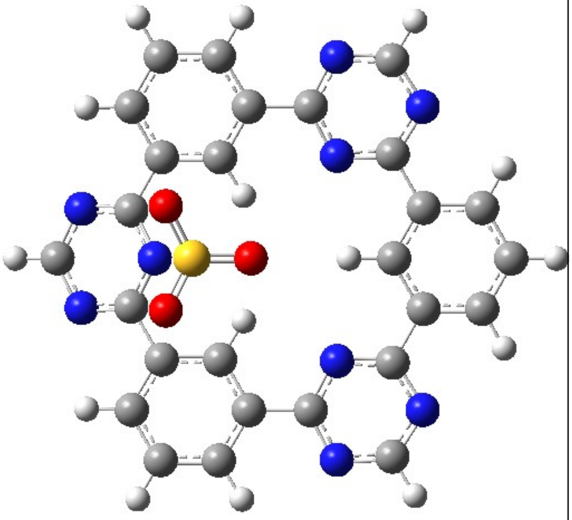<br>**-12.75 kcal/mol**	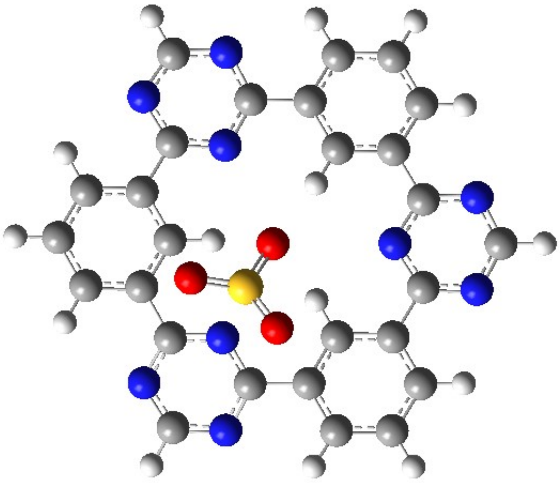<br>**-12.27kcal/mol**
**CO_2@CTF-0**	

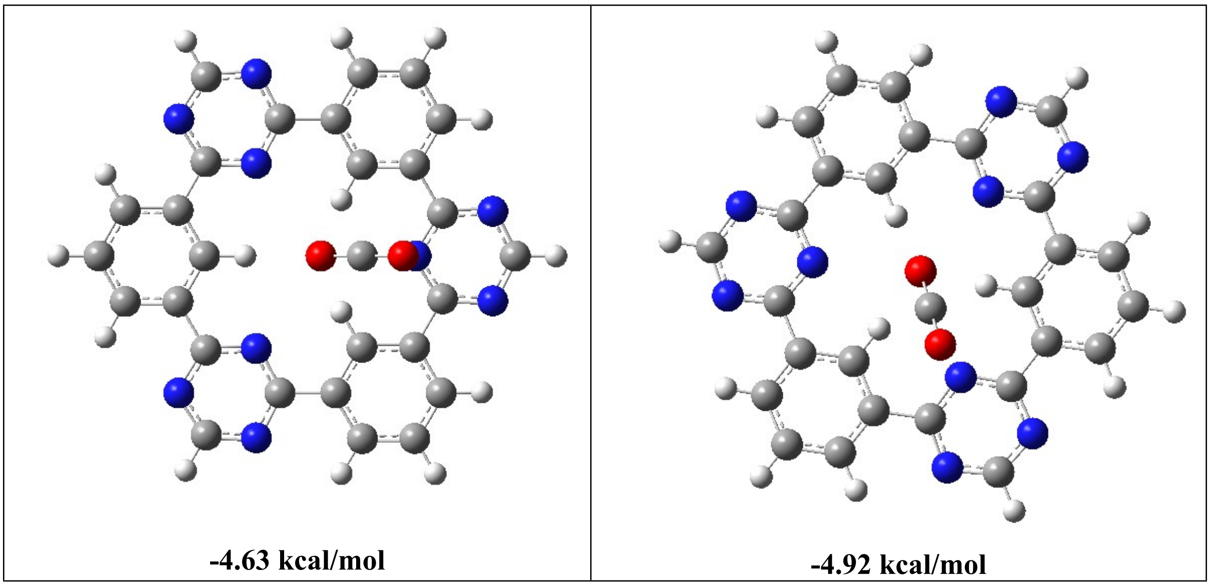

Figure S1: Possible orientations of studies analytes@CTF-0 complexes and their respective interaction energies

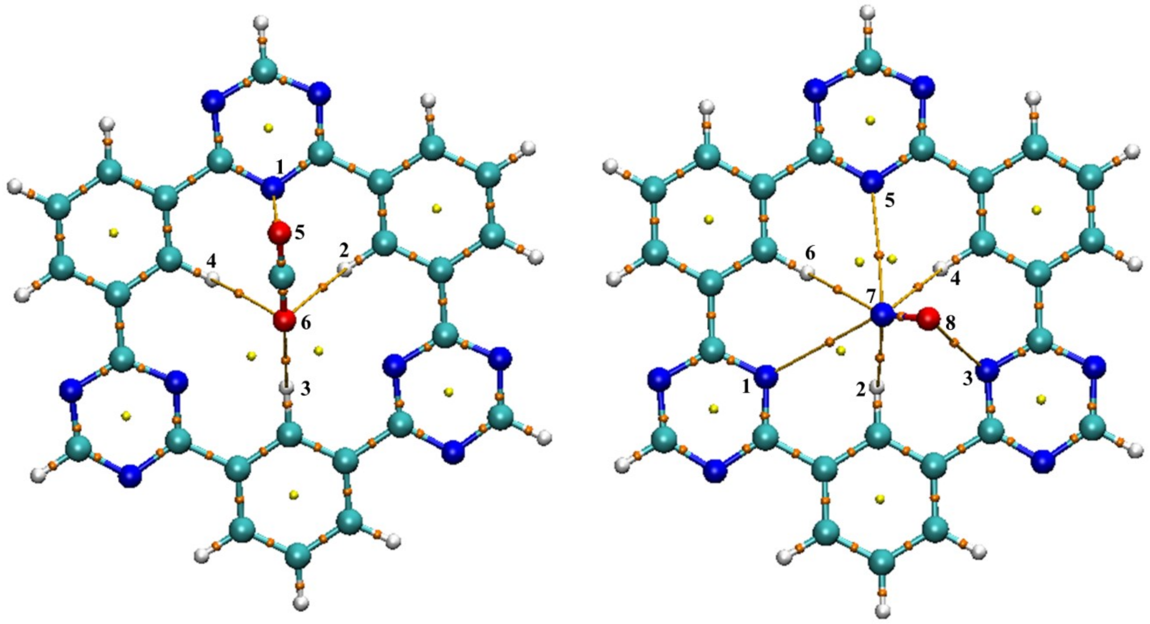

CO_2@CTF-0 **NO@CTF-0**

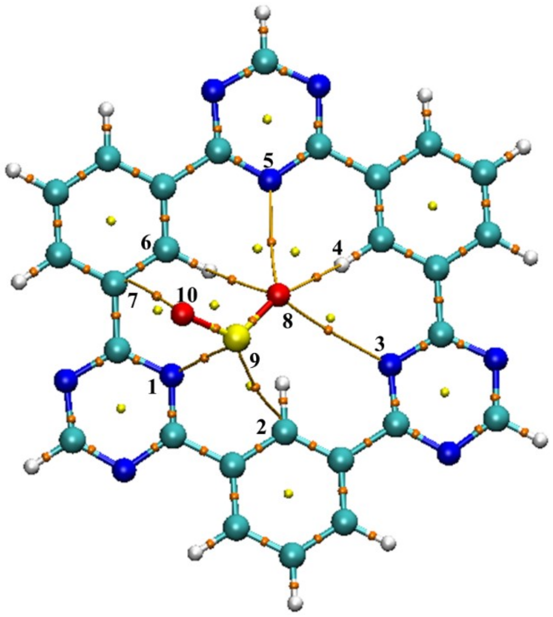

SO_2@CTF-0

Figure S2: QTAIM analysis results of CO_2@CTF-0, NO@CTF-0 and SO_2@CTF-0 complexes

Table S1: Recovery response time for complexes (analytes@CTF-0) at three different temperatures (298, 250 and 400 K).

Analytes@surface	Recovery Time (τ) seconds		
	298 K	350 K	400 K
O_3@CTF-0	9.72×10^{-09}	2.48×10^{-09}	9.35×10^{-10}
NO@CTF-0	4.74×10^{-10}	1.90×10^{-10}	9.85×10^{-11}
SO_2@CTF-0	1.47×10^{-07}	2.51×10^{-08}	7.08×10^{-09}
SO_3@CTF-0	3.39×10^{-03}	1.30×10^{-04}	1.26×10^{-05}
CO_2@CTF-0	4.87×10^{-09}	1.37×10^{-09}	5.58×10^{-10}

Cartesian Coordinates

CTF-0

0 1

C -5.46961000 0.00000200 0.00000000

N -4.87004300 -1.18444300 0.00000000

N -4.87004300 1.18444700 0.00000000

C	-3.53155400	-1.13180500	0.00000000
C	-3.53155400	1.13180800	0.00000000
N	-2.82614600	0.00000100	0.00000000
C	-2.78900100	-2.41411100	0.00000000
C	-3.48662400	-3.62636200	0.00000000
C	-1.39408300	-2.41362200	0.00000000
C	-2.78854000	-4.82930800	0.00000000
C	-0.69666100	-3.62160000	0.00000000
C	-1.39754600	-4.83209200	0.00000000
C	0.78539400	-3.62334300	0.00000000
N	1.40890600	-4.80876100	0.00000000
N	1.41319500	-2.44678700	0.00000000
C	2.73454900	-4.73626300	0.00000000
C	2.74606500	-2.49196200	0.00000000
N	3.46087400	-3.62491200	0.00000000
C	3.48579900	-1.20796800	0.00000000
C	4.88459000	-1.20618300	0.00000000
C	2.78844800	0.00000000	0.00000000
C	5.57718300	-0.00000200	0.00000000
C	3.48580200	1.20796500	0.00000000
C	4.88459300	1.20617900	0.00000000
C	2.74606800	2.49196100	0.00000000
N	3.46087500	3.62491100	0.00000000
N	1.41319700	2.44678600	0.00000000
C	2.73454900	4.73626300	0.00000000
C	0.78539600	3.62334000	0.00000000
N	1.40890700	4.80876000	0.00000000
C	-0.69665900	3.62159900	0.00000000
C	-1.39754200	4.83209300	0.00000000
C	-1.39408200	2.41362200	0.00000000

C	-2.78853600	4.82931000	0.00000000
C	-2.78900100	2.41411200	0.00000000
C	-3.48662100	3.62636500	0.00000000
H	-6.55445300	0.00000200	0.00000000
H	-4.56799900	-3.60936700	0.00000000
H	-0.85496600	-1.47764800	0.00000000
H	-3.32949500	-5.76680000	0.00000000
H	-0.84276300	-5.76030100	0.00000000
H	3.27631800	-5.67599400	0.00000000
H	5.41071700	-2.15097600	0.00000000
H	1.70819100	0.00000200	0.00000000
H	6.65962800	-0.00000600	0.00000000
H	5.41071900	2.15097300	0.00000000
H	3.27631900	5.67599300	0.00000000
H	-0.84275700	5.76030000	0.00000000
H	-0.85496500	1.47764800	0.00000000
H	-3.32949200	5.76680100	0.00000000
H	-4.56799700	3.60937200	0.00000000

O_3@CTF-0

0 1

C	-4.67833600	-2.60557900	-0.52645400
N	-3.58766800	-3.35955500	-0.45312600
N	-4.72027000	-1.27903600	-0.50312100
C	-2.44228100	-2.67511400	-0.34296500
C	-3.52539500	-0.68564300	-0.38742700
N	-2.36706800	-1.34329600	-0.30270000
C	-1.18041800	-3.44666200	-0.25696200
C	-1.21763200	-4.84490100	-0.22595700

C	0.04536800	-2.78190100	-0.21368800
C	-0.03330700	-5.56955600	-0.14811300
C	1.23155500	-3.51238500	-0.14045700
C	1.19055300	-4.90969100	-0.10621600
C	2.53518000	-2.80781600	-0.10442400
N	3.64650300	-3.55206900	-0.03422200
N	2.52538700	-1.47555300	-0.14604700
C	4.77632500	-2.85465700	-0.01075700
C	3.71718600	-0.87807800	-0.11134700
N	4.88452300	-1.53209900	-0.04530800
C	3.75395800	0.60255700	-0.14617600
C	4.98205400	1.27194200	-0.14819900
C	2.56358000	1.32958100	-0.17013600
C	5.01469900	2.66222600	-0.17441500
C	2.60018100	2.72380100	-0.19362300
C	3.82980900	3.39014900	-0.19484100
C	1.33803300	3.49970000	-0.20489000
N	1.42531600	4.83579400	-0.16087400
N	0.18874100	2.82509100	-0.25210300
C	0.25725800	5.46641900	-0.15933500
C	-0.92346900	3.56007700	-0.23397200
N	-0.94232300	4.89850000	-0.18840000
C	-2.22502600	2.85216700	-0.26021800
C	-3.41848800	3.57810400	-0.20756700
C	-2.26026100	1.45959900	-0.32879100
C	-4.63966300	2.91115600	-0.22178400
C	-3.48622300	0.79317100	-0.33694900
C	-4.67870800	1.52296400	-0.28571300
H	-5.62694100	-3.12393300	-0.61563500
H	-2.17545700	-5.34547100	-0.26399100

H	0.08092400	-1.70233100	-0.24132200
H	-0.06402600	-6.65124400	-0.12216200
H	2.11980400	-5.45995500	-0.04864600
H	5.70023000	-3.42042600	0.04325500
H	5.89574100	0.69379900	-0.12794800
H	1.61481100	0.81336700	-0.16281300
H	5.96557500	3.17925400	-0.17677500
H	3.84015800	4.47146100	-0.21053500
H	0.28585300	6.55041700	-0.12821500
H	-3.37350300	4.65743000	-0.15466200
H	-1.33544400	0.90284000	-0.38194000
H	-5.56243000	3.47525700	-0.17919300
H	-5.62022500	0.99122400	-0.29375100
O	-1.15613100	-0.44546400	2.17730200
O	-0.17580200	0.22316600	1.82402700
O	-2.15260000	0.16405700	2.58790800

NO@CTF-0

0 2

C	-0.04612900	5.47122900	-0.16549900
N	1.13982500	4.87465700	-0.17468600
N	-1.22887500	4.86885100	-0.14662400
C	1.09047400	3.53624000	-0.15820300
C	-1.17345900	3.53049300	-0.13187100
N	-0.03945900	2.82771700	-0.13247500
C	2.37432300	2.79673000	-0.16564800
C	3.58523800	3.49650500	-0.14696000
C	2.37619300	1.40208700	-0.18756900
C	4.78925700	2.80046800	-0.14633800

C	3.58524900	0.70684300	-0.18191900
C	4.79437000	1.40948600	-0.16203900
C	3.58857600	-0.77486800	-0.18903200
N	4.77479300	-1.39660500	-0.19613000
N	2.41255100	-1.40431300	-0.18377700
C	4.70452600	-2.72227400	-0.19668300
C	2.46044600	-2.73706000	-0.18261900
N	3.59431900	-3.45021100	-0.18919700
C	1.17775800	-3.47912500	-0.16736900
C	1.17930800	-4.87779300	-0.14939600
C	-0.03188300	-2.78431500	-0.16622800
C	-0.02501800	-5.57315000	-0.12898200
C	-1.23792200	-3.48491600	-0.14111800
C	-1.23283700	-4.88341100	-0.12355500
C	-2.52350700	-2.74782200	-0.12837400
N	-3.65475900	-3.46496900	-0.11499900
N	-2.47994000	-1.41508400	-0.12743800
C	-4.76727900	-2.74047800	-0.09960200
C	-3.65765900	-0.78931400	-0.10999500
N	-4.84189100	-1.41507800	-0.09547900
C	-3.65838100	0.69237700	-0.10531800
C	-4.86969800	1.39127100	-0.08396600
C	-2.45098600	1.39066700	-0.12086300
C	-4.86891900	2.78224500	-0.07821700
C	-2.45396300	2.78565400	-0.11437700
C	-3.66698400	3.48174000	-0.09342000
H	-0.04891200	6.55600200	-0.17592700
H	3.56614400	4.57773800	-0.13192700
H	1.44145900	0.86080700	-0.20690500
H	5.72580100	3.34282400	-0.13110500

H	5.72341000	0.85612200	-0.15798900
H	5.64505100	-3.26257400	-0.20354500
H	2.12540700	-5.40150000	-0.15128000
H	-0.03577800	-1.70383900	-0.18523200
H	-0.02230100	-6.65550900	-0.11552700
H	-2.17637100	-5.41145000	-0.10498800
H	-5.70602600	-3.28382300	-0.08906300
H	-5.79694500	0.83501200	-0.07187000
H	-1.51524900	0.85158500	-0.13530300
H	-5.80697300	3.32194900	-0.06146500
H	-3.65137100	4.56311500	-0.08929500
N	0.04235600	0.00048000	1.99481600
O	1.03170500	-0.08770500	2.56553700

SO_2@CTF-0

0 1

C	1.49198500	5.31192400	-0.04752300
N	0.19704000	5.02145700	-0.09341000
N	2.49469200	4.44312400	-0.09195600
C	-0.07473200	3.71534300	-0.21029600
C	2.11798100	3.16227900	-0.20366900
N	0.84996000	2.75822100	-0.27872500
C	-1.49770400	3.30655100	-0.26836100
C	-2.51055200	4.26680900	-0.19341300
C	-1.82579300	1.95750000	-0.39427400
C	-3.84520000	3.87681000	-0.24906600
C	-3.16468400	1.56928400	-0.44384300
C	-4.17664400	2.53315000	-0.37760100
C	-3.51569400	0.14093900	-0.57160400

N	-4.77936500	-0.17510900	-0.87966900
N	-2.55083000	-0.76425100	-0.36828500
C	-5.01797000	-1.47627900	-0.98551400
C	-2.89324500	-2.04398700	-0.54410900
N	-4.12844500	-2.45436100	-0.85038300
C	-1.84025900	-3.07035300	-0.37854500
C	-2.19032300	-4.42107800	-0.27689600
C	-0.49771800	-2.69278700	-0.33316600
C	-1.19929100	-5.38345100	-0.11766900
C	0.49363000	-3.66162800	-0.17818800
C	0.14015300	-5.00964100	-0.06799900
C	1.91980200	-3.25730600	-0.13680400
N	2.84097600	-4.22276200	-0.03094100
N	2.19607500	-1.95622800	-0.20887700
C	4.09498200	-3.78404700	-0.00878400
C	3.48842300	-1.62727600	-0.16532400
N	4.48581600	-2.51748800	-0.07056400
C	3.84302700	-0.19136800	-0.22480500
C	5.18587600	0.19274200	-0.30249600
C	2.83934200	0.77742100	-0.19622800
C	5.51972600	1.54161900	-0.35449700
C	3.17938300	2.12949400	-0.24488800
C	4.52265700	2.51073900	-0.32215900
H	1.75591900	6.36058100	0.03852800
H	-2.23796300	5.30860800	-0.09231100
H	-1.03442700	1.22427300	-0.45926900
H	-4.62785500	4.62203700	-0.18921200
H	-5.20926100	2.21462100	-0.42180200
H	-6.03664500	-1.77023100	-1.21396900
H	-3.23485400	-4.69832900	-0.32376000

H	-0.21529800	-1.65364800	-0.43308300
H	-1.47097600	-6.42770100	-0.03258300
H	0.92153600	-5.74790200	0.05137800
H	4.87454700	-4.53427500	0.06871500
H	5.95083900	-0.57175500	-0.32038300
H	1.80285500	0.48286300	-0.11506900
H	6.55872700	1.83847000	-0.41834800
H	4.76780600	3.56371200	-0.35487700
S	-1.33178000	-0.47991300	2.19139500
O	-0.08473100	0.17739300	1.83628900
O	-2.41787500	0.35993800	2.66992400

SO_3@CTF-0

0 1

C	-4.98062900	0.00029000	-1.38924500
N	-4.41023100	-1.18187800	-1.19176400
N	-4.41009100	1.18240100	-1.19172400
C	-3.22369400	-1.15018300	-0.58590300
C	-3.22356100	1.15055300	-0.58589900
N	-2.66326800	0.00014000	-0.15190000
C	-2.49604700	-2.41601300	-0.42488200
C	-3.20800300	-3.61957100	-0.37924100
C	-1.10219300	-2.41753300	-0.37491200
C	-2.52047800	-4.81998300	-0.25552100
C	-0.41770100	-3.62534800	-0.25878700
C	-1.12964400	-4.82639000	-0.19498600
C	1.06338400	-3.62859800	-0.19506200
N	1.68227000	-4.81125700	-0.09809300
N	1.68726500	-2.45246100	-0.23605900

C	3.00728200	-4.73471500	-0.04072500
C	3.01802500	-2.49209200	-0.15642900
N	3.73114800	-3.62249400	-0.06040800
C	3.75415900	-1.20723400	-0.17663100
C	5.15248100	-1.20576500	-0.21261400
C	3.05426600	-0.00017000	-0.15496600
C	5.84563800	-0.00033900	-0.23173600
C	3.75430400	1.20681000	-0.17664500
C	5.15262700	1.20517500	-0.21263900
C	3.01831900	2.49175800	-0.15648200
N	3.73159000	3.62210800	-0.06081000
N	1.68755200	2.45226100	-0.23586700
C	3.00785300	4.73440300	-0.04118100
C	1.06380600	3.62848400	-0.19502600
N	1.68283200	4.81108400	-0.09836800
C	-0.41728400	3.62538000	-0.25864400
C	-1.12909800	4.82649300	-0.19471400
C	-1.10191000	2.41764900	-0.37484200
C	-2.51993500	4.82024100	-0.25520100
C	-2.49576700	2.41628700	-0.42479000
C	-3.20759300	3.61991400	-0.37902000
H	-5.98935600	0.00035900	-1.78649600
H	-4.28817700	-3.59709400	-0.42928000
H	-0.53999600	-1.49554600	-0.42409400
H	-3.06704200	-5.75248000	-0.20388800
H	-0.58196700	-5.75417600	-0.10043800
H	3.54914800	-5.67149800	0.03199300
H	5.67818100	-2.15073400	-0.22409000
H	1.97435600	-0.00011000	-0.10210800
H	6.92766000	-0.00041000	-0.26115100

H	5.67843200	2.15008400	-0.22414300
H	3.54982400	5.67114000	0.03134000
H	-0.58132000	5.75421300	-0.10010600
H	-0.53983200	1.49559400	-0.42411700
H	-3.06639600	5.75279200	-0.20345700
H	-4.28777100	3.59755700	-0.42904500
S	-1.77677100	0.00010400	1.85224100
O	-2.39953500	1.24466700	2.25352100
O	-2.39974500	-1.24435800	2.25350900
O	-0.36728400	-0.00002200	1.51903400

CO_2@CTF-0

0 1

C	-2.84920700	-4.73676200	-0.08056800
N	-1.52381000	-4.81110500	-0.11496200
N	-3.57422000	-3.62485800	-0.08436400
C	-0.90151100	-3.62631200	-0.16303600
C	-2.85953800	-2.49244200	-0.13161900
N	-1.52728900	-2.44945700	-0.17616700
C	0.58040500	-3.62439600	-0.20173100
C	1.28459400	-4.83175600	-0.16157800
C	1.27373100	-2.41614700	-0.27597300
C	2.67548500	-4.82609400	-0.19446500
C	2.66864300	-2.41370900	-0.30634700
C	3.36994700	-3.62354000	-0.26737800
C	3.40772000	-1.13188400	-0.38123500
N	4.73896600	-1.18379600	-0.51097700
N	2.70524600	0.00036900	-0.30835400
C	5.33597500	0.00079600	-0.56384300

C	3.40736100	1.13285400	-0.38113800
N	4.73858600	1.18519500	-0.51087500
C	2.66788900	2.41444900	-0.30621300
C	3.36884800	3.62447600	-0.26722300
C	1.27297400	2.41650100	-0.27587600
C	2.67404900	4.82683500	-0.19431300
C	0.57930800	3.62456300	-0.20166300
C	1.28315700	4.83211500	-0.16147100
C	-0.90261400	3.62606300	-0.16308000
N	-1.52527400	4.81066900	-0.11512500
N	-1.52803900	2.44902300	-0.17618900
C	-2.85065500	4.73592600	-0.08083400
C	-2.86030500	2.49160100	-0.13171900
N	-3.57533300	3.62380300	-0.08460600
C	-3.59805000	1.20691100	-0.13422600
C	-4.99683100	1.20505900	-0.13653800
C	-2.89900700	-0.00042500	-0.13146500
C	-5.68963300	-0.00084800	-0.13836800
C	-3.59767800	-1.20797800	-0.13416600
C	-4.99646300	-1.20654600	-0.13647600
H	-3.39140900	-5.67553900	-0.04436600
H	0.73258800	-5.75994000	-0.10476300
H	0.72705600	-1.48507800	-0.31581000
H	3.21915100	-5.76145600	-0.16229700
H	4.45090500	-3.60446700	-0.29409400
H	6.41608400	0.00097000	-0.66369500
H	4.44981200	3.60571600	-0.29391700
H	0.72654600	1.48528000	-0.31572500
H	3.21745500	5.76234800	-0.16211500
H	0.73088200	5.76014000	-0.10468100

H	-3.39314500	5.67453900	-0.04471100
H	-5.52292000	2.14985700	-0.13656700
H	-1.81865100	-0.00024300	-0.11905100
H	-6.77206600	-0.00101300	-0.14107000
H	-5.52226200	-2.15150500	-0.13645500
C	1.28274900	0.00025600	2.34710400
O	0.18868900	-0.00016900	1.95342700
O	2.36882300	0.00068000	2.76448100
